# Application and Potential of Local Drug Delivery Systems for Antibacterial Treatment of Periodontitis

**DOI:** 10.3390/ijms27072983

**Published:** 2026-03-25

**Authors:** Xinchao Wang, Fengli Wu, Jia Liu, Xingqi Hong, Shujun Dong

**Affiliations:** Hospital of Stomatology, Jilin University, Changchun 130012, China; xinchao23@mails.jlu.edu.cn (X.W.); wufl23@mails.jlu.edu.cn (F.W.); jialiu24@mails.jlu.edu.cn (J.L.); xqhong24@mails.jlu.edu.cn (X.H.)

**Keywords:** local drug delivery systems, nanotechnology, anti-microbial therapy, periodontitis, chronic inflammation

## Abstract

Periodontitis (PD) is a chronic inflammatory disease characterized by the progressive destruction of periodontal supporting tissues. As one of the most prevalent chronic diseases, PD affects more than 743 million people globally, some with serious systemic health implications. Plaque accumulation constitutes the key driver of periodontitis, initiating host inflammatory cascades and compromising periodontal microbiome equilibrium. Conventional treatment methods, such as scaling and root planing, are limited by a constrained operative field, resulting in blind spots that impede the complete eradication of bacterial biofilms and the modulation of the inflammatory microenvironment. Therefore, employing new therapeutic strategies (e.g., drug delivery systems) is essential. This review focuses on local drug delivery systems for the treatment of PD, including fibers, strips and films, microspheres, gels, nanoparticles, and vesicle systems, to deliver drugs directly into the periodontal pockets, targeting inflammation and providing sustained antibacterial effects while reducing systemic side effects. The characteristics and clinical implications of each type of local drug delivery system are discussed, along with emerging technologies such as 3D printing and nanotechnology.

## 1. Introduction

Periodontitis (PD) is a chronic inflammatory disease that causes the progressive destruction of periodontal tissues [1,2]. PD is a multifactorial disease involving genetic susceptibility [3], environmental factors [4], and an oral microbiota imbalance [5,6]. The formation of periodontal pockets due to plaque accumulation provides an environment for the growth of pathogenic microorganisms, exacerbating PD and complicating its treatment [7,8]. Dental plaque is the primary initiating factor [9,10]. Consequently, antimicrobial therapy constitutes a critical component of PD treatment research. While traditional mechanical treatments (e.g., supragingival scaling and root planning) can remove some plaque, they are ineffective at reaching complex periodontal pockets and root bifurcations and cannot remove endotoxins that have penetrated the root surface [11,12]. Conventional local drug delivery methods for PD primarily include mouthwash and locally applied agents. Although mouthrinses such as chlorhexidine (CHX) can enhance therapeutic efficacy, their simple local administration and short oral residence time hinder the sustained maintenance of an effective antibacterial concentration [13]. Furthermore, a higher concentration of CHX may induce adverse effects, including taste alteration, oral mucosal desquamation, and tooth/tongue discoloration [14]. As the most widely used local drug for periodontitis in current clinical practice, minocycline hydrochloride offers three primary advantages: broad-spectrum antibacterial activity with low resistance rates, promotion of periodontal tissue regeneration, and modulation of the host microenvironment. However, its clinical application is limited by unstable release kinetics requiring frequent administration and high treatment costs [15]. Consequently, the development of new therapeutic strategies for PD is paramount.

**Figure 1:**
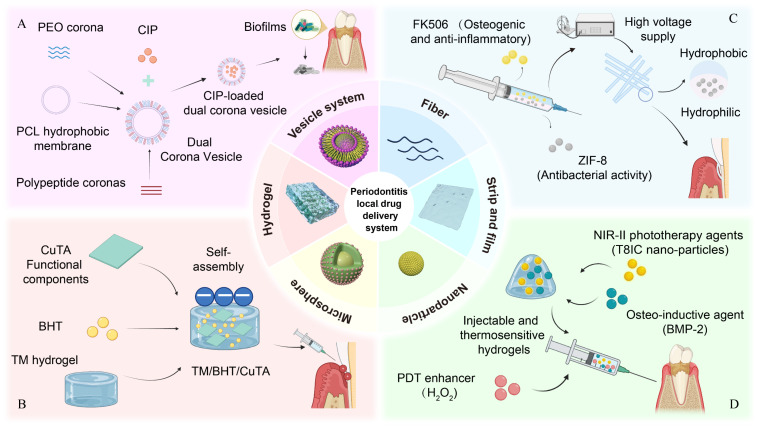
Local drug delivery systems in the management of periodontitis and representative examples. (**A**) Schematic illustration of a multifunctional dual-corona polymer vesicle that facilitates biofilm penetration and enhances antibiotic delivery. (**B**) Matrix metalloproteinase (MMP)-responsive TM/BHT/CuTA hydrogel enabling on-demand release of CuTA nanozymes with antibacterial, ROS-scavenging, and immunomodulatory functions. (**C**) Janus nanofibers with biphasic release behavior enabling rapid antibacterial action and sustained osteogenic stimulation for guided tissue regeneration. (**D**) Injectable thermosensitive hydrogel co-delivering BMP-2 and NIR-II phototherapy agents (T8IC) for synergistic antibacterial therapy and bone regeneration. This illustration was created by the authors based on information summarized from the cited studies.

Local drug delivery systems have been recognized as a potential alternative treatment for PD. Local drug delivery systems can directly target the infection site, reduce systemic side effects, prolong the drug half-life, maintain the long-term effective drug concentrations in the long term, and achieve sustained antibacterial effects [16]. Local drug delivery systems vehicles, such as fibers, strips and films, microspheres, gels, nanoparticles, and vesicle systems, have been designed to meet varying patient needs (Figure 1). This review describes local drug delivery systems applications in anti-bacterial treatment for PD, including their material composition, processing methodologies, distinctive innovations, and clinical implications. We also discuss emerging trends, such as hydrogels that have the potential to revolutionize PD management. We summarize how local drug delivery systems can be used to transform the treatment of PD, providing clinicians with a new tool to combat this global health problem.

## 2. Challenges of Antibacterial Therapy in Periodontitis

The growing concern regarding antimicrobial resistance (AMR) presents a significant challenge in managing periodontitis. Numerous periodontal pathogens have demonstrated reduced susceptibility to commonly prescribed antibiotics, potentially undermining the effectiveness of traditional antimicrobial therapies [17,18]. Furthermore, the dysbiotic polymicrobial community and the highly structured extracellular polymeric matrix of periodontal biofilms collectively restrict drug diffusion and enhance bacterial tolerance [19], making biofilm-associated infections particularly difficult to eradicate [20,21]. These limitations highlight the urgent need for alternative antibacterial strategies and improved therapeutic approaches for effective periodontal therapy. Recently, increasing attention has been directed toward the development of alternative antimicrobial agents and advanced therapeutic strategies, such as metal-based materials [22] and natural bioactive compounds [23]. In particular, these delivery systems have been developed to overcome biofilm-associated barriers by improving local drug retention [24,25], facilitating penetration into biofilms [26], and enabling controlled or stimuli-responsive drug release [27]. Therefore, advanced drug delivery systems have emerged as promising platforms for enhancing antibacterial therapy against complex periodontal biofilms. These platforms may significantly improve the therapeutic efficacy of antibacterial agents by enhancing drug concentration at the infection site while minimizing systemic exposure.

## 3. Drug Delivery System for PD Treatment

Both systemic drug therapy and local drug administration are important components of therapy for PD [28]. Systemic antibiotic therapy is limited by its inability to consistently achieve effective concentrations in the periodontal pocket and by its frequent side effects, such as allergic reactions, gastrointestinal disturbances, and the promotion of antibiotic resistance [16]. Conversely, local drug administration achieves high effective drug concentrations through direct application to the target site, thereby minimizing systemic side effects [29]. However, due to salivary clearance and daily oral hygiene, the drugs lack sufficient retention time to completely eradicate the biofilm. Therefore, there is an urgent need to develop novel local drug delivery system technologies for the treatment of PD. Local drug delivery systems can be directly applied into the periodontal pocket, enabling sustained and controlled release of drug. Local drug delivery systems present several advantages, including minimal tissue invasiveness, reduced dosage, decreased administration frequency, and a consequent reduction in adverse effects. The reduced pain and convenience associated with local drug delivery systems also enhance patient compliance.

Beyond these practical advantages, these systems exert their therapeutic efficacy through multiple interrelated material and structural design mechanisms. The selection of polymers, crosslinking density, and scaffold architecture can be optimized to modulate degradation rates and drug release profiles, thereby facilitating sustained and predictable delivery of therapeutics [30,31]. Structural designs, such as fibers and hydrogels, enhance mechanical stability, minimize premature drug washout, and ensure localized drug availability [25,32]. The incorporation of natural polymers or bioactive derivatives confers biological advantages by improving biocompatibility, offering intrinsic anti-inflammatory or antimicrobial properties, and promoting periodontal tissue regeneration [33,34]. Together, these mechanisms translate into clinical benefits such as minimally invasive placement, reduced dosing frequency, and enhanced patient compliance compared with conventional mechanical periodontal therapies.

Despite the promising therapeutic potential demonstrated in numerous in vitro and preclinical studies, the clinical translation of many novel local drug delivery systems remains limited. Challenges, including manufacturing complexity, regulatory requirements, long-term biosafety evaluations, and the absence of well-designed large-scale clinical trials, continue to restrict their widespread clinical adoption [35]. Consequently, additional well-designed clinical studies are necessary to systematically evaluate the safety, efficacy, and long-term therapeutic outcomes of these emerging biomaterial-based delivery platforms in the treatment of periodontitis [36,37]. This highlights the need to design local drug delivery systems that not only exhibit efficacy in vitro and in vivo but also possess features that enhance clinical translation. An ideal local drug delivery system should be biodegradable, biocompatible, and easy to place, it should also exhibit long-term stability to ensure sustained drug delivery and must not affect oral appearance [38]. To date, the main forms of local drug delivery systems include fibers, strips and films, microspheres, gels, nanoparticles, and vesicle systems [39]. The advantages and limitations of some common local drug delivery systems for the treatment of PD, as well as commonly used polymers in local drug delivery systems and their key properties, are summarized in Table 1 and Table 2.

## 4. Existing and Developing Local Drug Delivery Systems for PD

### 4.1. Fiber

Fibers are linear or spiral-shaped natural/synthetic substances with a hollow structure that can be used to store and release drugs. In PD treatment, fiber delivery system is commonly placed into the periodontal pocket, by winding it around the tooth root. The system is then secured with periodontal dressing or cyanoacrylate adhesive [54] to hold the fibers in place. In 1979, Goodson et al. [55] observed that tetracycline-loaded cellulose acetate hollow fibers inhibited periodontal flora at concentration 1000 times lower than systemic doses. However, this drug delivery system failed to provide controlled drug release, as the hollow fibers released the drug too rapidly. Consequently, subsequent studies focused on developing matrix fibers from polymers (e.g., polyurethane, polypropylene, cellulose acetate propionate, and ethyl vinyl acetate). The manufacturing process involved mixing drugs with a molten polymer and then spinning it into fibers upon cooling [56]. The development of a tetracycline-loaded ethyl acetate vinyl fiber was prepared, demonstrating sustained release for up to 9 days in vitro [56]. However, this drug delivery system was non-biodegradable. Major disadvantages of the clinical application of fibers for PD treatment include the complicated operation process of fiber placement, the need for removal of the fibers, and an increased number of patient visits, all of which will cause physical and psychological discomfort to the patient and reduce patient compliance. In addition, fibers may also cause local gingival redness, which further limits their application [57]. Biodegradable natural/synthetic substances, such as zein, chitosan (CS), nylon, PCL, alginate, and collagen, were subsequently developed and have gradually been used in the manufacture of fibers [38,58].

In recent years, nanofibers (typically 50–500 nm in diameter) have been employed to overcome some limitations of fibers. Their key advantages for local drug delivery systems applications include simulation/restoration of extracellular matrix, high biocompatibility, superior mechanical strength, tunable porosity, and a high surface area-to-volume ratio [59,60]. Nanofibers can be fabricated by electrospinning, phase separation, self-assembly, and laser spinning [58]. And nanofibers can be composed of natural, synthetic, and mixed polymers, such as ceramics, metal compounds, PCL, poly(p-dioxanone), and CS, improving their tolerance and stability [61]. The application of nanofibers in the antimicrobial therapy of PD has attracted extensive attention due to their excellent drug-carrying properties. Dennis et al. [62] prepared a polylactic acid (PLA) electrospun fiber loaded with a complex antibacterial agent composed of ampicillin and metronidazole (MNZ), which showed significant inhibitory effects on PD pathogens *A. actinomycetemcomitans*, *P. gingivalis*, *Fusobacterium nucleatum*, and *Enterococcus faecalis*. In addition, PCL was also loaded with the two antibacterial agents oxytetracycline hydrochloride and zinc oxide for the treatment of PD [43]. In vitro studies exhibited that the drug could be released continuously for up to 10 h and showed excellent activity against mixed bacterial cultures. Furthermore, CS, possessing good bioadhesion and antimicrobial properties, serves as an excipient to maintain the morphological stability of nanofibers and enhance their adhesion in periodontal pockets. In 2019, a double-layer nanofiber mat consisting of a CS/poly(ethylene oxide) (PEO) nanofiber layer and a PCL nanofiber layer [63] was reported, with 30% ciprofloxacin in the CS/PEO layer and 5% MNZ in the caprolactone layer. Ciprofloxacin exhibited a slow release, attributable to its low solubility at pH 7.4 and the presence of solid drugs in the delivery system. MNZ achieved controlled release through PCL barrier, across which it slowly diffused into the surrounding medium. This double-layer material demonstrated antibacterial activity against *Escherichia coli* and *A. actinomycetemcomitans*, and sustained drug release for over 7 days.

As an advancement in electrospinning technology, two-fluid electrospinning is now widely used in medical research. Danilo et al. [64] fabricated bilayer zein-based membranes using a combination of coaxial electrospinning and 3D printing to accelerate periodontal tissue regeneration. The drug delivery system consisted of a core-sheath type electrospun nanofiber—with a core of PEO/curcumin (Cur)/tetracycline hydrochloride and a sheath of zeolysin/PCL/β-glycerophosphate—which were deposited onto a 3D-printed honeycomb PLA/zein/Cur platform. This created a bilayer structure that simulated periodontal tissue. In vitro, the system demonstrated antibacterial activity against the red complex of periodontitis-causing bacteria and exhibited a sustained drug release profile lasting over 8 days. In addition, core-sheath electrospun nanofibers could be integrated with polymer micelles to create a time-programmable drug delivery system. This system was designed to simultaneously achieve anti-inflammatory activity and bone regeneration, addressing the critical challenge of alveolar bone resorption in PD treatment [65]. In line with the concept of multifunctional nanostructures, including core–shell designs, recent studies introduced nuclear–satellite nanoarchitectures (PPAg), which integrated Prussian blue with silver nanoparticles through a polydopamine bridge [66]. These composites exhibited synergistic antibacterial and reactive oxygen species (ROS)-scavenging effects, reduced pro-inflammatory cytokines, and facilitated periodontal tissue healing in vivo. Furthermore, core–shell nanofiber designs developed for pulp–dentin complex regeneration offered a conceptual framework for applying these multifunctional architectures to periodontal therapy, particularly due to their ability to enable compartmentalized loading and controlled release of multiple therapeutic agents [67]. Such designs may therefore be adapted for periodontal therapy by enabling spatially controlled delivery of antibacterial and regenerative agents.

Nanofibers can be further modified to enhance their mechanical properties, cell adhesion, and biocompatibility for application in PD treatment. Recent studies reported the development of electrospun fibrous scaffolds based on natural polymers, including methacrylated silk fibroin combined with cobalt-doped bioactive glass [34]. These scaffolds demonstrated inhibitory effects against biofilms of *Porphyromonas gingivalis* and exhibited favorable biocompatibility along with a minimal inflammatory response in vivo [34]. Such findings suggest their potential applications in infection control and periodontal tissue regeneration. Janus nanofibers with biphasic release properties have been developed to replace commercially available guided tissue regeneration membranes due to their lack of antimicrobial and osteogenic properties [44] (Figure 1). However, challenges such as high production costs and scalability limitations hinder their widespread clinical adoption [58], necessitating further research to overcome these barriers for efficient PD management.

### 4.2. Strips and Films

Strips and films (SFs) are thin-sheet drug delivery systems composed of polymers in which drugs are embedded or bound. Drug release occurs through diffusion and/or matrix dissolution/erosion, with rates modulated by the SFs’ size and targeted application [68]. The main advantages of SFs are their capacity to match the shape and size of the periodontal pocket, including larger ones. This custom fit enhances patient comfort and compliance [16,38]. SFs can be classified as non-degradable or degradable systems. Non-degradable SFs have a simple preparation process, while degradable SFs do not need a secondary removal operation, reducing patient revisits [39,69]. SFs can be composed of natural or synthetic biodegradable polymers, including CS, atelocollagen, poly(lactic-co-glycolic acid) (PLGA), gelatin, or polycaprolactone (PCL). Of these polymers, PCL is a common choice for formulating SFs. A D-optimal experimental design method was employed to optimize the PCL film formulation for controlled metronidazole (MNZ) release. The independent variables (MNZ, PCL, hydroxypropyl methylcellulose, and glycerol monooleate) were selected to successfully develop the film [70]. In the treatment of PD, therapeutic strategy has shifted from a single antibacterial treatment to combined antibacterial, anti-inflammatory, and tissue regeneration strategies. This shift has driven the development of multi-functional treatment. Hydroxypropyl methylcellulose, diclofenac, CHX/betamethasone, CHX/lidocaine, CHX/Carbopol 917, and polyethylene glycol (PEG) were employed to form a system of mucoadhesive films co-loaded with antimicrobial and anti-inflammatory agents; the system showed significant inhibition of bacterial growth and local inflammation [52]. A double-layer membrane system was developed to combine mechanical reinforcement with dual drug release: a Gellan gum layer loaded with moxifloxacin hydrochloride and a hydroxyethyl cellulose layer incorporated with clove essential oil. This membrane showed antibacterial activity against both Gram-positive bacteria (*Staphylococcus aureus*) and Gram-negative bacteria (*Escherichia coli*). The membrane employed a synergistic strategy by providing rapid release of clove oil for analgesic purposes alongside sustained release of moxifloxacin hydrochloride (over 48 h) for antibacterial action, thereby simultaneously managing the symptoms and root cause of periodontitis with considerable promise [71].

The development of environmentally responsive drug delivery systems is also a current research focus. These systems can control drug release in response to stimuli such as temperature, light, pH, reactive oxygen species (ROS), glucose, or enzymes. As a representative example, antibacterial photodynamic therapy (aPDT) employs photosensitizer drugs (PS) that, upon irradiation with a light source, activate molecular oxygen to generate ROS, thereby achieving a potent antibacterial effect. However, there are some factors that limit the clinical application of aPDT, such as inadequate oxygen concentrations in deep hypoxic pockets. To solve the problem, a novel thin-film drug delivery system was reported, which isolated the PS (chlorin e6) onto a solid superhydrophobic (SH) film composed of polydimethylsiloxane. The film featured a rough surface topography with microchannels that allow air to diffuse to the PS, thus ensuring a sufficient oxygen supply. This system addressed the issues of aPDT being affected in the anoxic environment of the periodontal pocket and provided a reference for the further application of aPDT [72].

Despite progress, clinical application of SFs has been limited by their poor retention in the periodontal pocket. To address this limitation, a new generation of nanostructured barrier membranes with enhanced properties is being developed that, in addition to providing local drug delivery, can promote cell adhesion, migration, proliferation, and differentiation, thereby promoting tissue regeneration [73].

### 4.3. Microspheres

Microspheres are solid spherical polymers with diameters ranging from 1 to 1000 μm, in which drugs are dispersed throughout the polymer matrix [74]. They can be classified into biodegradable and non-biodegradable types, based on natural/synthetic polymers. For administration, they can be incorporated into various forms such as gels, toothpaste, or even embedded in microchip-based delivery devices. The main advantages of microspheres include shielding of unstable drugs prior to and post-administration, controlled drug release, sustained therapeutic effects, enhanced bioavailability, and improved patient compliance [38,54]. To enhance doxycycline (DXY) encapsulation efficiency, Raghavendra et al. [75] employed PLGA and PCL to prepare sustained-release DXY microspheres, achieving a prolonged effective local concentration for up to 11 days. Improvement of the formulation parameters of the emulsification method (a common method for drug encapsulation in microspheres) was proposed to increase the encapsulation efficiency of DXY in microspheres [76]. The authors identified the optimal formulation ratio and stated that microspheres with higher drug loads exhibit lower drug release rates. This occurred because at higher drug load levels, stronger interactions between microspheres cause aggregation. These aggregations penetrate the matrix, which slows down drug dissolution and diffusion, thus reducing the release rate. This study confirmed the significant potential of DXY-loaded microspheres treating PD. Similarly, a pH-sensitive nano/microsphere system was developed by combining PLGA with CS, possessing pH-dependent dissolution characteristics. Metronidazole and N-phenacylthiazolium bromide (PTB) were encapsulated in PLGA nano/microspheres and coated with chitosan (CS). This system effectively preserved the antimicrobial activity of the drugs and provided a more sustained release profile compared to conventional hydrogel systems [77]. Aside from CS and PLGA, microspheres composed of other components are gaining widespread attention, including polyphosphates, especially calcium polyphosphate (CPP) glass, which has been experimentally proven to load minocycline and achieve sustained release for up to 7 days [78]. CPP exhibited excellent biocompatibility owing to its composition of bioactive calcium (Ca^2+^) and phosphate ions. This property, combined with its multifaceted efficacy (antibacterial, anti-inflammatory, and pro-osteogenic), made CPP a highly promising material for the clinical treatment of PD [78].

At present, despite extensive research on the use of microspheres, there are few clinical applications. Key challenges include: (i) insufficient drug loading requiring microsphere architecture modifications for controlled release profiles, and (ii) high manufacturing costs limiting clinical adoption [79]. Therefore, further research must focus on developing novel microsphere systems that exhibit high drug loading, minimal toxicity and side effects, ease of manufacture, and broad clinical utility.

### 4.4. Gels

Gels are semi-solid systems that enable the uniform distribution of drugs in a solid matrix, offering facile application and simple preparation for targeted periodontal drug delivery. Gels, which possess high biocompatibility and bioadhesive properties, can be better retained in situ than other materials, ensuring long-term stable drug release with a lower risk of irritant or allergic host reactions [80]. Gels for periodontal delivery are primarily composed of natural or synthetic polymers, such as carbomer, hydroxymethyl cellulose, and CS. Among various gel formulations, injectable in situ gels have gained significant attention. These systems exist as a sol (liquid) state in vitro but undergo sol-to-gel transition upon injection into the periodontal pocket. A novel in situ gel system, composed of a bleach shellac, DXY hydrochloride, MNZ, benzoyl peroxide (BP), and the solvent N-methyl-2-pyrrolidone, exhibited enhanced viscosity, while retaining antibacterial activity of DXY, MNZ, and BP. It extended drug release time to twice that of the pure drug, suggesting its strong potential for in situ gel systems in PD treatment [81].

Ionic liquids can also be used for gel treatment of PD. An iongel was prepared from a choline/geranic acid (1:2) deep eutectic antimicrobial (IDEA), with gelation induced by aqueous addition. This gel system exhibited high viscosity, capability for rapid penetration after periodontal injection, enhanced epithelial permeability, and antibacterial activity [82].

Natural drugs have been widely investigated for the treatment of PD in recent years due to their low toxicity, as well as their antibacterial and anti-inflammatory properties. Rajeshwari et al. [83] prepared a thermoreversible gel composed of green tea extract using Poloxamer 407, a heat-sensitive polymer, and Carbopol 934. The system exhibited strong bioadhesion, along with antibacterial and anti-inflammatory properties. It provided sustained drug release for over 96 h and demonstrated low cytotoxicity. This study showed the significant potential of combining natural medicines with thermoreversible gel systems for the clinical management of PD.

Hydrogels are hydrophilic polymer networks with a 3D cross-linked structure held together by particle interactions and hydrogen bonds. With the high water content of hydrogels (typically 70–99%), hydrogels exhibit physical similarities to biological tissues, resulting in excellent biocompatibility, and making them ideal matrices for encapsulating hydrophilic drugs. The versatile fabrication methods, variable composition, and extensive mechanical properties of hydrogels are all advantages of their application in PD treatment [54,84,85]. Injectable hydrogels, particularly thermosensitive hydrogels, can be used to treat complex and irregular periodontal pockets by in situ injection. These hydrogels can be formulated using various polymers, such as Pluronic, PLGA, collagen, and CS. Drug release from these systems is primarily controlled by the mesh size, degradation rate, swelling behavior, and mechanical properties [84]. Conventional hydrogels often suffer from a burst release of drugs. To address this limitation, environmentally responsive hydrogels that control drug release in response to specific microenvironment changes provide a new strategy for PD treatment. As an example, an ROS-responsive hydrogel was fabricated with oxidized dextran (OD) and phenylboronic acid-functionalized poly (ethylene imine) (PBA-PEI) through the in situ formation of Schiff base, loaded with DXY and metformin. This drug delivery system exhibited ROS responsiveness, strong adhesion, and drug synergy, showing considerable potential for the application of hydrogel systems in PD treatment [50].

**Figure 2:**
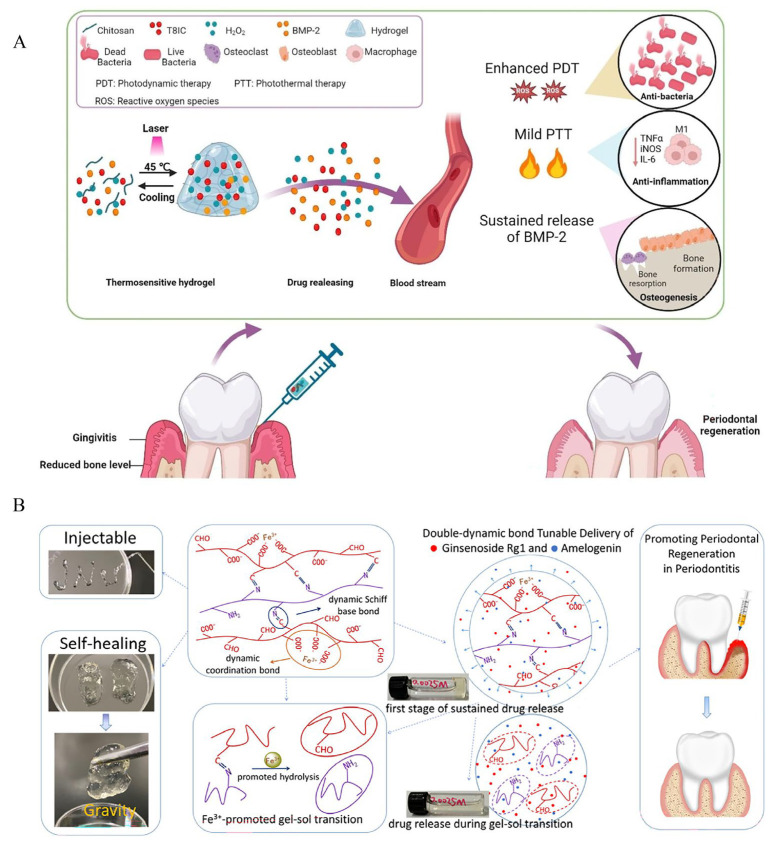
(**A**) Hydrogel composed of T8IC, H_2_O_2_, and BMP2 and its role in the treatment of periodontitis. Source: Reprinted with permission of the Creative Commons Attribution 4.0 from Ref. [47]. (**B**) Diagram of a double dynamic bond hydrogel and its drug release process. Source: Reprinted with permission from Ref. [51]. Copyright 2025, with permission from American Chemical Society.

Hydrogels can also be combined with antibacterial photothermal (aPTT)/photodynamic therapy (aPDT). In one study, researchers developed a charge-tuned enhanced Type I photosensitizer (PS), ethyl blue (EB), through multi-alkylation modification. The modified EB demonstrated approximately 2-fold higher absorbance and generates 2.2–4.2 times more superoxide anions (O_2_^−^) than conventional Type I PS, sulfur-substituted Nile blue (NBS). When loaded into an oxidized dextran(O-Dex)/quaternized chitosan (QCS) hydrogel, the PS achieved localized sustained release. This system enabled efficient bacterial inhibition under both red light and sunlight irradiation, even in hypoxic conditions [49]. This system combined photothermal and antibacterial effects, providing a new gel system for PD treatment. On this basis, hydrogels can also be combined with nanomaterials to more effectively control drug release and enhance antibacterial effects. An injectable, thermosensitive hydrogel composed of 1,2-distearoyl-sn-glycero-3-phosphoethanolamine-N-[methoxy(PEG)-2000] was developed to serve as a carrier to support bone morphogenetic protein 2 (BMP2), an NIR-II phototherapy agent (T8IC nanoparticles), and H_2_O_2_, in which H_2_O_2_ was used to promote the antibacterial effect of aPDT and BMP2 was used for osteogenesis. Consequently, this system achieved synergistic antibacterial, anti-inflammatory, and bone regeneration effects, demonstrating significant potential for comprehensive PD treatment [47] (Figure 2A).

Recent advances in 3D printing technologies utilizing hydrogels have enabled the fabrication of multifunctional scaffolds and drug delivery systems for periodontal regeneration. These platforms allow precise spatial organization of biomaterials, bioactive molecules, and cells, thereby supporting the repair of both soft and hard tissues. For instance, a 3D-printed bilayer hydrogel incorporating lipid nanoparticles loaded with grape seed extract and simvastatin demonstrated synergistic antibacterial activity and osteogenic potential, enabling sustained drug release and improved periodontal regeneration [86]. In addition, various bioink strategies have been explored to target specific tissues. For hard tissue regeneration, EphrinB2-overexpressing dental pulp stem cells (DPSCs) embedded in low-concentration GelMA hydrogels were bioprinted into 3D constructs, where the cells maintained high viability and exhibited enhanced osteogenic differentiation, accompanied by upregulated markers such as ALP, BMP2, RUNX2, and SP7 [87]. For soft tissue repair, micro-extruded acellular dermal matrix (ADM)/gelatin/alginate hydrogels encapsulating gingival fibroblasts were bioprinted into scaffolds that significantly improved keratinized gingival augmentation and enhanced tissue integration in vivo [88].

In general, gel systems play a major role in the treatment of PD, with intelligent reactive hydrogels and nano-hydrogels providing a new path for PD treatment. Moreover, as an effective treatment strategy for PD, a variety of synergistic drug-carrying gels have great development prospects and are worthy of further study. However, the clinical translation remains challenging due to limitations of hydrogels, including the inefficient synthesis and gelation processes, time-consuming production, and high manufacturing costs, which collectively hinder large-scale production.

### 4.5. Nanoparticles

Nanoparticles provide a novel strategy for the antimicrobial treatment of PD due to their inherent advantages, such as ultra-small size, large surface area to volume ratio, which can increase the amount of loaded ions, strong adsorption, structural stability, and targeting ability [89,90]. In addition, the particular structure and physical properties of nanoparticles can achieve antibacterial and anti-inflammatory activities simultaneously [91], demonstrating significant potential for PD treatment.

#### 4.5.1. Polymer Nanoparticles

Compared with metal nanoparticles, polymer nanoparticles exhibit superior biodegradability, greater stability, favorable physical and chemical properties, and improved biocompatibility, making them an integral part of PD antibacterial treatment of local drug delivery systems [92]. Polymer nanoparticles mainly carry drugs through dissolution, encapsulation, entrapment, or attachment to the nanoparticle matrix. Their high dispersibility in aqueous media enables stable drug release and enhances system stability. Furthermore, the special chemical structures of polymer nanoparticles permit covalently binding of functional groups to the target sites, simultaneous incorporation of both hydrophilic and hydrophobic substances, and adaptability to diverse drug administration routes [93]. These nanoparticles can be fabricated from either natural or synthetic polymers, among which CS and PLGA are the most widely used.

As a natural polymer, CS nanoparticles (CSNPs) have excellent degradability, biocompatibility, antibacterial property, tissue healing property, and osteoinductive potential [94]. CSNPs have a cationic global charge and can interact with negatively charged bacterial membranes to achieve bactericidal effects [95]. To overcome the limited solubility of CS in water, O-substituted carboxymethyl groups can be introduced to enhance its solubility at neutral and alkaline pH. On this basis, a tetracycline encapsulated O-carboxymethyl CSNP was developed. This system demonstrated good biocompatibility and achieved an effective intracellular drug concentration for bactericidal activity, thereby enhancing its antibacterial efficacy [96]. In addition, CSNPs can be combined with other nanomaterials to achieve synergies. For instance, CS-coated ZnO nanoparticles exhibited enhanced antimicrobial activity, antioxidant properties, and mechanical strength compared to neat CSNPs. One key mechanism involved the release of zinc ions (Zn^2+^), which differs from the antibacterial action of CSNPs. Zn^2+^ induced mitochondrial weakness, and restriction of cellular growth and integrity by the leakage of lipids, proteins and DNA, ultimately leading to cellular lethality [97]. Compared to the neat polyvinyl alcohol (PVA) hydrogel, the CSNPs/PVA composite hydrogel exhibited improved blood compatibility, enhanced antibacterial activity, and superior mechanical strength. Furthermore, the incorporation of tetracycline significantly increased its hydrophilicity [98]. A drug delivery system composed of CSNP and PLGA nanoparticles achieved sequential release of multiple drugs. For instance, PLGA–lovastatin–CS–tetracycline nanoparticles achieved sequential release of tetracycline and lovastatin, leading to simultaneous antibacterial and osteogenic effects [99].

PLGA provides optimal mechanical properties and the capacity to load both hydrophilic and hydrophobic molecules. It is widely regarded as one of the most biocompatible and degradable materials [38]. A major limitation of PLGA is its low encapsulation efficiency for hydrophilic compounds, frequently resulting in an initial burst release. To overcome this, Tahereh et al. [100] prepared a minocycline-PLGA nanoparticle system by ion coupling method, which achieved superior drug loading, encapsulation efficiency, and antibacterial activity compared to standard formulations, demonstrating great promise for treating PD. With the CS concentration increased, both the particle size and the drug encapsulation efficiency of CS-PLGA nanoparticles also increased. This modification effectively reduced the initial burst release and concurrently improved the antibacterial activity of the drug [101]. In addition, PLGA nanoparticles can also bind to peptides. Mohamed et al. [102] modified the surface of PLGA nanoparticles with peptides from *Streptococcus gordonii*, which resulted in significantly more effective biofilm disruption compared to free peptides. Furthermore, aPDT was combined with PLGA nanoparticles loaded methylene blue (MB). This combination enhanced the permeability of the photosensitizer within both the tissue and the biofilm matrix. Consequently, it significantly improved the efficacy of aPDT, ultimately achieving control of PD [103]. Thus, the combination of nanoparticles and aPDT has great potential in the clinical application of PD treatment.

In general, polymer nanoparticles demonstrate strong potential for the antimicrobial therapy of PD. However, their clinical application faces several challenges, including complex synthesis processes and high manufacturing costs.

#### 4.5.2. Metal Nanoparticles

Metals and their oxides are important components of nanostructured antibacterial materials. Their nanoparticles mediate multiple antibacterial mechanisms, including electrostatic adsorption to the cell wall, disruption of bacterial membrane, induction of oxidative stress, and binding to intracellular components [104]. Some metal nanoparticles can also be applied to aPTT/aPDT because of their photoactivity [105]. Owing to their multiple antibacterial mechanisms, metal nanoparticles pose a low risk of bacterial resistance, offering a promising alternative treatment method. These nanoparticles also exhibit excellent chemical stability, catalytic activity, and therapeutic selectivity, suggesting great potential in the research of PD treatment. The antibacterial effect is strongly influenced by their morphology and size, with sub-10 nm particles exhibiting optimal activity. It likely results from induced changes in the local electronic structure of the bacterial surface and smaller particles have a higher contact area with bacteria [106]. To date, the most extensively studied metal nanoparticles include silver nanoparticles (AgNPs), zinc and zinc oxide nanomaterials, nano-copper, titanium dioxide nanomaterials, and cerium and cerium oxides.

Because of their broad-spectrum antibacterial activity, ultra-small size, and high contact area, AgNPs are considered one of the most promising nanomaterials for the antibacterial treatment of PD. AgNPs exert their antibacterial effect mainly through direct membrane destruction, affecting mitochondrial function, binding with DNA to affect replication, releasing a large amount of Ag^+^, and producing high ROS concentrations [107]. The antibacterial effect of AgNPs can be modulated by their size and shape due to the difference in specific surface area [108]. Generally, the antibacterial effect of AgNPs diminishes with increasing particle size [106], and studies have shown that triangular AgNPs may have higher antibacterial activity than spherical or rod-shaped AgNPs [109]. In 2013, Besinis et al. [110] prepared an AgNP coating directly applied to the dentin surface and confirmed its antibacterial activity.

**Figure 3:**
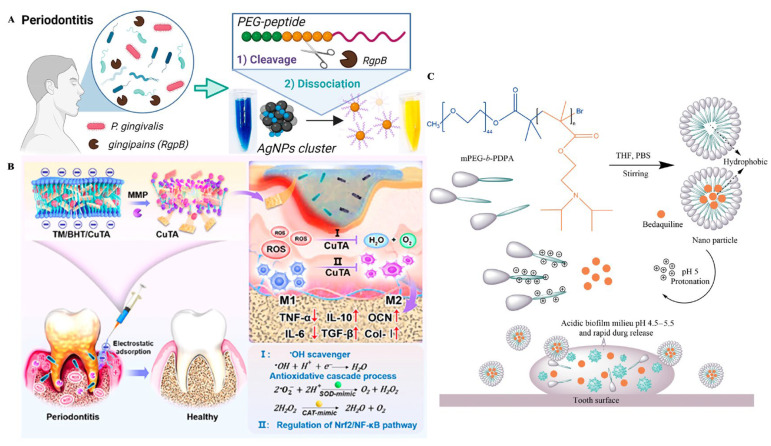
(**A**) Schematic illustration of peptide–PEG functionalized silver nanoparticle assemblies. Source: Reprinted with permission from Ref. [114]. Copyright 2026, with permission from American Chemical Society. (**B**) Schematic illustration of the TM/BHT/CuTA hydrogel system for enzyme-responsive retention and on-demand release of CuTA nanozymes, enabling antibacterial and antibiofilm activity in periodontitis. Source: Reprinted with permission from Ref. [91]. Copyright 2026, with permission from American Chemical Society. (**C**) Schematic representation for the formation and pH-responsive property of the nanomicelle. Source: Reprinted with permission of the Creative Commons CC-BY from Ref. [117].

Additionally, CHX/MNZ was conjugated onto AgNPs, in which CHX was directly conjugated with AgNPs and MNZ was connected to the AgNPs surface by a PEG joint. In vitro experiments demonstrated that the complex has effective antibacterial and anti-inflammatory properties [111]. AgNPs can also be combined with natural/synthetic polymer nanoparticles to integrate the high antibacterial activity of AgNPs with the hydrophilicity and biocompatibility of natural polymers and the superior mechanical and biological properties of synthetic polymers. On this basis, a PLGA/CS/Ag nanocomplex was developed with simultaneous antibacterial activity, promotion of cell mineralization, and excellent biocompatibility at the optimal ratio of the three substances [112]. Furthermore, AgNPs can be combined with peptides to increase their stability and biocompatibility. Zorraquín-Peña et al. [113] developed novel glutathione-stabilized AgNPs, which retained the antibacterial activity of nano-Ag while significantly improving the biocompatibility. Additionally, a reversible aggregation of AgNPs was reported using a cationic glycine-based peptide and sulfur-terminated PEG [114] (Figure 3A). This coupling can detect *P. gingivalis* enzyme activity, providing a new strategy for the study of antimicrobial therapy for PD. AgNPs can also be combined with sonodynamic therapy (SDT). A novel nano-acoustic sensitizer composed of AgNPs, titanium dioxide, silicon dioxide, and quaternary ammonium CS was developed, demonstrating potent antibacterial and anti-inflammatory effects [115]. However, the primary concerns of AgNPs involve biosafety due to accumulation in the internal organs, causing liver and kidney damage when used in large quantities, and the risk of release into the environment [116].

Zinc and its oxide nanoparticles also demonstrate significant antimicrobial efficacy in PD therapy. Nano-zinc mainly exerts its antibacterial effect through direct interaction with the -NH_2_ and -SH functional groups of proteins, while ZnO nanomaterials generate ROS and Zn^2+^ through oxygen defect points. These agents interfere with DNA replication, metabolism, and change membrane permeability to achieve antibacterial effect [118]. ZnO is photoactive, which can generate a large number of hydroxyl radicals (-OH), hydrogen peroxide (H_2_O_2_), and superoxide free radicals (•O_2_^−^) upon light irradiation, which can initiate redox reactions of other drugs in the system [119]. Furthermore, the advantages of zinc and its oxide nanomaterials lie in their role as essential human trace elements, their potential to enhance angiogenesis and osteogenesis, their broad antibacterial spectrum, and their high stability [104]. The antibacterial properties of zinc and its oxide nanomaterials are also related to their size and shape, with enhanced activity at smaller particle sizes. A flexible PCL matrix was employed as a carrier of ZnO nanoparticles, creating a multifunctional membrane that exploits both the osteogenic and antibacterial properties of ZnO [120]. To overcome the limited antibacterial spectrum of ZnO nanoparticles and their reliance on high concentrations for specific bacteria, AgNPs were introduced and Ag/ZnO nanocomposites were fabricated by sedimentation and precipitation method, with the advantages of enhanced antibacterial activity and excellent biocompatibility [121]. Additionally, the photoactivity of ZnO enables its application in aPTT/aPDT. For example, a composite of ZnO nanowires and PDA-black phosphorus nanosheets on a titanium substrate exhibited an enhanced photothermal effect under NIR irradiation. The photothermal reaction of black phosphorus nanosheets can also accelerate the release of Zn^+^, significantly boosting antibacterial activity [122]. At present, the main difficulty in the clinical application of zinc and its oxide nanomaterials is that their long-term biocompatibility and metabolic matrix are unclear [123].

As an essential trace element like zinc, copper nanoparticles (Cu NPs) possess multiple bioactive properties including antibacterial effects, angiogenesis promotion, and osteogenic enhancement [104]. Cu NPs exert antibacterial effects by inducing oxidative stress, which causes bacterial cell membrane damage and inhibits bacterial cell metabolism [124]. However, at high concentrations, Cu NPs can induce toxicity in humans, including gastrointestinal bleeding, liver and kidney damage, and nerve damage [125]. To reduce these side effects, a copper ion polymer hydrogel was synthesized by chelating poly(acrylic acid-coitaconic acid) with copper ions [126]. This system chelated Cu^2+^, reducing free ion concentration and preventing toxic accumulation in normal cells. It provided targeted, controlled release to bacteria, avoiding local over-concentration and minimizing toxicity. Furthermore, the system was biodegradable and demonstrated excellent biosafety. Moreover, a continuous release drug system can be designed to achieve the long-term release of Cu^2+^ at biocompatible concentrations. A zinc-based zeolite-imidazolate framework loaded with copper ions (Cu@ZIF-8) was synthesized. A blend of PCL/PLA/nano-hydroxyapatite was then grafted onto this framework to guide bone regeneration [127]. This composite material exhibited both antibacterial and osteogenic effects, providing a novel strategy for the treatment of PD bone defects.

With the development of nanomaterials, environmentally responsive materials have also become a focus of research. A self-assembly system was developed, composed of the copper-based nanozyme (copper tannic acid coordination nanosheets) and a triglycerol monostearate/2,6-di-tert-butyl-4-methylphenol hydrogel, which hydrolyzed with the increase in matrix metalloproteinases (Figure 3B). The self-assembly system utilized its inherent anionic charge for electrostatic targeting and robust adhesion to the positively charged inflammatory microenvironment. This mechanism circumvented rapid salivary clearance and secured extended nanozyme retention (over 7 days) within the periodontal pocket. The resulting spatial confinement of therapy augmented local bioavailability while mitigating systemic exposure [91]. Copper nanoparticles can also be applied to aPDT. Kong et al. [128] developed a novel heterogeneous nanocomposite, Bi_2_S_3_/Cu-tetrakis(4-carboxyphenyl)porphyrins (Bi_2_S_3_/Cu-TCPP). The photothermal effect of Bi_2_S_3_ accelerated the release of Cu^+^ ions. This process achieves effective antibacterial activity, reduces inflammation, and promotes bone protection.

Other metal and metal oxide nanoparticles, including gold, calcium-based, cerium dioxide (CeO_2_), and titanium dioxide, have also been extensively researched. Among them, nano-titanium dioxide, nano-gold, nano-cerium, nano-iron, and their oxide nanoparticles can be combined with aPDT to enhance their antibacterial activity. For example, titanium dioxide nanoparticles (TiO_2_ NPs) can produce ROS and hydroxyl radicals under ultraviolet irradiation, and these active substances can effectively inactivate bacteria [129]. Notably, iron and its oxide nanomaterials, as essential trace elements for the human body, are the active sites of many electron transport enzymes and oxygen transport enzymes, while demonstrating excellent biocompatibility [130,131]. As a component element of bone, calcium has the potential to promote bone formation and mineralization. CaSiO_3_ nanoparticles, amorphous calcium phosphate nanoparticles, and nano-hydroxyapatite (nHA) exhibit antibacterial activity alongside significant potential for osteogenesis and mineralization [132,133]. The clinical application of this field in PD treatment is forthcoming [134,135]. Because of the advantages including high specific surface area, excellent chemical stability, and small size, metal nanoparticles show considerable potential for clinical application in PD treatment. However, concerns regarding their long-term safety profile, biocompatibility, and antibacterial efficacy must be addressed through further research.

#### 4.5.3. Nanometer Micelles

Nanomicelles are ordered supramolecular structures composed of amphiphilic molecules self-assembled in aqueous solution. These nanostructures are mainly divided into self-assembled nanomicelles and polymer nanomicelles. They begin to self-assemble by increasing the surfactant to a specific concentration, known as the critical micelle concentration. Surfactants are amphiphilic molecules with a hydrophilic head and a hydrophobic tail. Micellar size can be controlled by head group and alkyl chain length [136,137]. Nanomicelles with a low critical micelle concentration are suitable for local drug delivery systems due to their high stability and insensitivity to dilution, enabling prolonged local retention [138]. Most polymer nanomicelles have a core–shell structure, wherein the shell is composed of hydrophilic blocks, which mainly control the pharmacokinetic properties in vivo, while the core is composed of hydrophobic nuclei, mainly responsible for drug inclusion, controlled drug release properties, and the stability of nanomicelles. The main advantages of nanomicelles are their small size, good biocompatibility, and the ability to enclose hydrophobic drugs in their cores to increase drug availability [139,140]. Moreover, compared with other delivery systems, nanomicelles exhibit lower dissociation kinetics after dilution [139,141]. Natural polyphenol chlorogenic acid (CGA) was loaded onto PLGA and modified with polyvinylpyrrolidone. The obtained CGA-PLGA@polyvinylpyrrolidone nanomicelles showed effective slow release of CGA and effective ROS, thereby controlling the inflammatory response of PD [142]. Similarly, a pH-responsive nanomicelle composed of methoxypolyethylene glycol-b-poly-2-(diisopropyl amino) ethyl methacrylate (mPEG-b-PDPA) and loaded with bedaquiline was developed. This system demonstrated potent bactericidal activity against *Streptococcus mutans* and effectively disrupted mature biofilms without inducing cytotoxicity [117] (Figure 3C). However, the long-term side effects of nanomicelles are unknown, and experiments are still needed to prove their clinical availability [143].

#### 4.5.4. Other Nanoparticles

In addition to polymer and metal nanoparticles, such as mesoporous silica nanoparticles (MSNs), are also promising nanocarriers due to their tunable morphology, mesoscopic structure and porosity, high biocompatibility, functional simplicity, high surface area, and large pore volumes enhancing drug-loading capacity and delivery efficiency [144]. Moreover, MSNs can release drugs at specific sites in response to external stimuli, and can be combined with different drugs to improve their functionality [145]. The antibacterial performance of MSNs is related to their shape. Experiments have shown that CHX-loaded spherical MSNs has larger surface area, higher CHX release rate, and greater anti-biofilm efficiency than linear MSNs [146]. However, MSNs also have shortcomings, such as potential cytotoxicity, uncertain metabolism, and complex synthesis process, which will affect their further clinical application in PD treatment.

The primary nanoparticles utilized in periodontal local drug delivery can be summarized in terms of their respective strengths and limitations. Polymer nanoparticles are characterized by their biodegradability, favorable biocompatibility, and sustained drug release; however, their complex synthesis and high costs may impede clinical application [92]. Metal nanoparticles provide rapid antibacterial effects through multiple mechanisms, yet they raise concerns about cytotoxicity and long-term safety [104,105]. Other nanoparticles, such as MSNs, facilitate high drug loading and stimulus-responsive release, although challenges persist due to their fabrication complexity and uncertain metabolic pathways [145]. These comparisons underscore the complementary features of these nanoparticles, which can inform the rational design of local drug delivery systems.

### 4.6. Vesicle System

#### 4.6.1. Liposomes

Liposomes are nearly spherical vesicular structures composed of natural or synthetic phospholipid bilayers. Liposomes used for medical purposes typically range in size from 50 to 450 nm [147]. These structures offer significant advantages for drug delivery, including versatility, targeting, tissue permeability, biodegradability, non-toxicity, and non-immunogenicity [74]. Liposomal drug delivery can be either active or passive. In the passive mode, hydrophilic drugs are distributed in the aqueous phase while hydrophobic drugs are encapsulated within the double layer of liposomes. In contrast, the active method, also known as remote loading, utilized transmembrane pH and ion gradients to drive drugs through the lipid bilayer, achieving higher encapsulation efficiency [94,148]. Drug release rates can be controlled by improving the properties of the liposomes by incorporating other substances, such as cholesterol, to reduce the permeability of the liposomes, increase their stability in vivo and in vitro, and induce the dense accumulation of phospholipids [149]. An alternative strategy for controlling drug release is choosing drugs that are easy to retain in the liposomes. Drugs have different retention properties in the liposomes due to their different physical properties such as their permeability, which is low for hydrophilic drugs and high for hydrophobic drugs. Furthermore, controlled release of drugs can be enhanced by loading concentrations of drugs that exceed the solubility limit in high liposomes to enhance precipitation [150,151]. The physical and chemical properties of liposomes affect their efficacy in preventing and removing biofilms—their size, size distribution, double-layer characteristics, surface characteristics, and high drug encapsulation rate play an important role in the effective delivery of antimicrobials to biofilms [152]. Studies have shown that the bacterial adsorption degree of cationic liposomes is higher than that of anionic liposomes of the same size range [153]. These critical properties, size, composition, charge, and layered structure of liposomes can be modulated by changing the preparation method and lipid components [54].

The trigger mechanism of liposomes refers to the stimuli that induce the release of their contents upon reaching the target site. These triggers include both external stimuli (e.g., light, heat, ultrasound) and intrinsic microenvironmental conditions (e.g., enzyme and pH changes) in diseased tissues or organelles. To date, the most common stimuli utilized for PD treatment are changes in pH and light (for aPDT). For example, pH-responsive N,N,N-trimethyl–CS (TMC) liposomes (TMC-lip) were developed to deliver doxycycline (DXY) [154]. TMC, the simplest form of quaternary ammonium CS, carried a permanent positive charge. In the acidic environment created by bacterial metabolism of the periodontal pocket, TMC underwent further protonation. This increased the surface positive charge, destabilized the nanoparticles, and triggered the release of encapsulated DXY [154]. In addition, to integrate liposomes with aPDT agent, a liposomal system co-encapsulating Cur and DXY (NL-Cur+DXY) was developed. The antibacterial activity and antimetabolic activity of aPDT mediated by this system were tested; the results showed that it could effectively reduce biofilm formation and metabolic activity. Furthermore, liposomes can also be combined with lipid gel. Methylene blue (MB) and bioactive glass were embedded in the precursor of a lipid gel; under 660 nm light, MB produced ROS, thus achieving an antibacterial effect, with great potential for its clinical application in PD treatment [155].

However, liposomes also have some disadvantages such as limited stability, drug leakage, fusion, high production costs, and a short circulatory half-life [156]. Fortunately, these limitations can be mitigated by modifying vesicle composition. For example, modification with PEG prolongs circulation time of liposomes, and the conjugation of targeting ligands, such as antibodies or lectins, facilitates specific cellular targeting [157,158]. To enhance membrane permeability, a submicron-sized liposomal drug delivery system was developed, featuring PEG-stabilized membranes and internally synthesized magnetite nanoparticles [159].

#### 4.6.2. Bacterial Outer Membrane Vesicles and Exosomes

**Figure 4:**
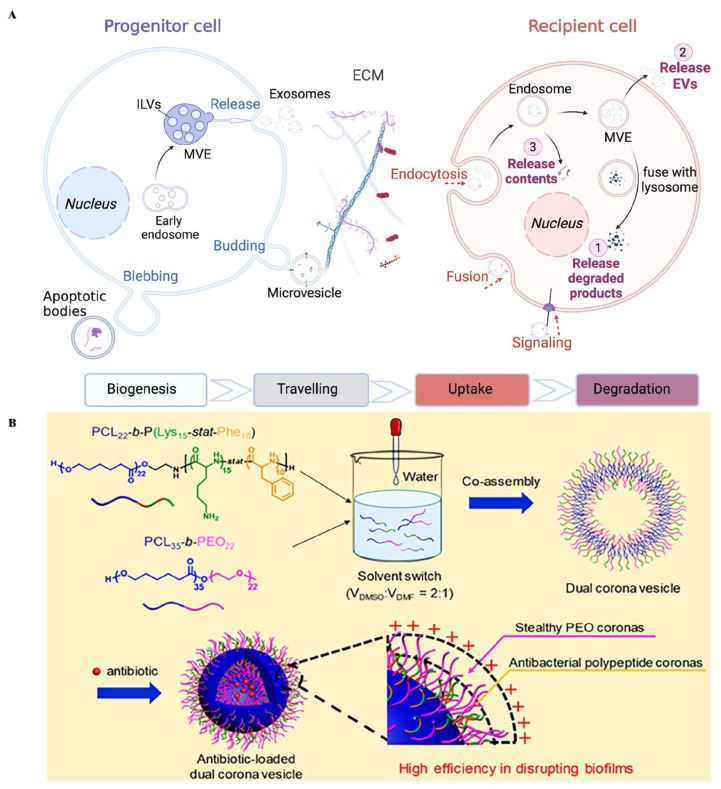
(**A**) The biogenesis, traveling, uptake and degradation of EVs. The arrows indicate the direction and progression of the processes, while the colors distinguish EV secretion and degradation pathways, and the numbers correspond to the three possible intracellular fates of EVs in recipient cells. (Abbreviation: ECM, extracellular matrix; MVE, multivesicular vesicle; ILVs, intraluminal vesicles) Source: Reprinted with permission of the Creative Commons CC-BY from Ref. [161]. (**B**) Preparation and synergistic antibacterial effect of PCL22-b-P(Lys15-stat-Phe10) and PEO22-b-PCL35 copolymers. Source: Reprinted with permission from Ref. [93]. Copyright 2025, with permission from American Chemical Society.

Extracellular vesicles (EVs), comprising exosomes, microvesicles, and apoptotic bodies, are cell-released, lipid-bilayer that are primarily categorized by the mode of biogenesis [160,161] (Figure 4A). Exosomes, with a diameter of 40 to 100 nm, are capable of encapsulating a variety of biomolecules within their lumen or embedded in their membrane. Exosomes are an important medium of intercellular communication [162]. Their contents are dependent on the phenotype of their donor cells. This property allows exosomes to be engineered into highly specific drug delivery systems by choosing an appropriate cellular source. A notable example is the use of exosomes derived from periodontal ligament stem cells (PDLSC-Exos) [163]. They promoted bone and periodontal regeneration as well as angiogenesis by enhancing cellular proliferation, promoting osteogenic differentiation, modulating miRNA expression, and activating signaling pathways. Concurrently, these exosomes exerted anti-inflammatory effects through regulation of inflammatory cytokines and macrophage polarization. The structure of exosomes is similar to that of liposomes, and the drug-loading mechanism also involves encapsulating hydrophilic drugs within the core, while incorporating hydrophobic drugs into the lipid bilayer. Drugs can be preloaded before exosome separation or loaded after exosome separation [160]. Exosome pre-isolation loading refers to the incorporation of drugs into exosomes during their biogenesis. However, since the drug encapsulation mechanism during exosome biogenesis is still unclear, controlling drug encapsulation efficiency remains a challenge. Consequently, several post-isolation loading methods have been developed, including direct co-incubation, sonication, electroporation, and freeze–thaw cycles [164]. In addition to direct co-incubation, which is the simple incorporation of drugs into purified exosomes, other methods are mainly used to change, destroy tight lipid structures or recombine lipid structures by mechanical force or electricity to improve drug encapsulation efficiency [164,165]. There are three main types of drugs delivered by exosomes: small- and medium-molecule drugs, nucleic acids, and proteins and peptides [166]. Kojic acid (KA) is a natural small-molecule compound derived from fungi that functions as a sonosensitizer to enhance the efficacy of antimicrobial SDT. Antimicrobial SDT has promising applications in destroying microbial biofilms; however, KA is unstable and induces side effects at high concentrations. In order to overcome these adverse effects, exosomes derived from periodontal stem cells were used to envelop KA. This approach significantly improved the stability, solubility, targeting capability, and efficacy of KA, while simultaneously reducing its toxicity [167]. Furthermore, the system effectively inhibited biofilm formation and increased ROS production. However, despite these promising results, several challenges persist in the application of exosomes including high production costs, demanding storage conditions, short biological activity, the absence of separation and purification standards, and medical ethical issues [160,168].

Bacterial outer membrane vesicles (OMVs) are natural functional nanomaterials secreted by Gram-negative bacteria. Their structure is analogous to that of exosomes. Furthermore, OMVs play crucial roles in bacterial communication, biofilm formation, and pathogenic processes [169]. In terms of antibacterial activity, OMVs can assist Gram-negative bacteria in inhibiting the growth of competitors. For instance, it was first reported that *Pseudomonas aeruginosa* produces OMVs carrying internal autolysins [170]. Autolysins are intracellular peptidoglycan (PG) hydrolases, and PG is a key structural component of the cell wall. OMVs deliver autolysins to other bacteria, leading to cell wall disintegration through PG hydrolysis in both Gram-positive and Gram-negative bacteria. Additionally, OMVs affect biofilm formation, mainly through blocking adhesion-driven biofilm formation and promoting bacterial movement and whole-body migration [171]. The main advantages of OMVs as a drug delivery system are high drug loading capacity, targeting ability, and thermal stability [172,173]. Studies have shown that the use of gentamicin interference can make the outer membrane of *P. aeruginosa* unstable, thus generating membrane vesicles and forming pores on the surface of the cell membrane [174,175]. This method can also increase OMVs production in other Gram-negative bacteria, facilitating the preparation of gentamicin–OMV complexes, which exhibit stronger antibacterial activity against both Gram-negative and intracellular infections [176].

Accurate targeting ability is a crucial criterion for evaluating novel drug carriers. Modifying OMVs represents a promising strategy to improve their targeting specificity. OMVs can be engineered through genetic approaches, such as transfecting bacteria with plasmid DNA to express specific ligands [177,178]. However, the therapeutic application of OMVs faces several challenges. These include the environmental susceptibility of the load content, which directly influences antibacterial activity; poor stability under physiological conditions; difficulties in controlling drug release kinetics, and an incomplete understanding of their potential pathogenicity. Further research is needed to address these limitations and optimize OMVs for clinical use.

#### 4.6.3. Polymer Vesicles

Polymer vesicles have been widely used in various biomedical fields due to their hollow nanostructures and different functions. They comprise a hydrophobic membrane and a hydrophilic inner nanoscale hollow sphere, creating a more complex structure than the single shell–core structure of nanomicelles, and their membrane is similar to the cell membrane composed of lipid bilayer [179,180]. Compared with liposomes, polymer vesicles exhibit greater chemical diversity, simpler preparation and functionalization processes, and enhanced stability and robustness [181]. The endocytosis process of polymer vesicles can aid in clearing pathogens from cells, which is difficult with antibiotics alone, and promoting the internalization of polymer vesicles enhances antibacterial activity [182]. Antimicrobial agents are usually bound to polymer vesicles in two mechanisms: deposited on the membrane of the polymer vesicles or encapsulated in the inner pore/membrane of the polymer vesicle. This integration offers several advantages, including modulating the surface charge density of the vesicles, prolonging the circulation time in vivo, reducing the cytotoxicity of antibacterial agents, and enabling on-demand drug release for multifunctional therapeutic applications. For instance, AgNPs can be deposited onto polymer vesicles, which not only prevents their agglomeration but also enhances antibacterial efficacy, showing great potential for the treatment of PD [183,184]. Antibiotics are usually encapsulated in the internal pores or membranes of polymer vesicles to achieve the required release at specific sites. For example, Kornchanok et al. [185] encapsulated MNZ/DXY in polymer vesicles; treatment with encapsulated MNZ/DXY led to a significant reduction in the intracellular number of *P. gingivalis* compared with free MNZ/DXY or separate polymer vesicles. Furthermore, penicillin-carrying polymer vesicles were grafted into a hydrogel network via Schiff base bonds to obtain the penicillin-loaded polymer vesicle–hydrogel composite [186]. The hydrogel facilitated an initial rapid release of penicillin for immediate antibacterial effects, followed by a sustained release of both the remaining penicillin and the polymer vesicles themselves for over 72 h. Moreover, the surface charge of the polymer vesicles also influences their antibacterial efficacy. Positively charged polymer vesicles can interact with negatively charged bacterial cell membranes, leading to membrane disruption. Multifunctional vesicles exhibiting intrinsic antibacterial and antibiotic-enhancing activities were developed through the co-assembly of two functional block copolymers with different functions [93]: PCL-block-poly(lysine-stat-phenylalanine) and poly(ethylene oxide)-block-PCL. The PCL chains of the two copolymers formed the membrane of the polymer vesicles. The poly(ethylene oxide) crown exhibited protein-repellent properties and demonstrated penetration capability through the extracellular polymeric substances (EPS) barrier of biofilms (invisibility). Meanwhile, the antibacterial peptide PCL-block-poly(lysine-stat-phenylalanine) made the surface of the polymer vesicles positively charged, promoting bacterial adhesion and subsequent membrane disturbance. Multi-functional applications of enhanced antibacterial activity and ‘invisibility’ are realized [93] (Figure 4B).

At present, research is intensely focused on manipulating both the membrane structure and the physicochemical properties of the corona in polymer vesicles. The adjustment of membrane structure can regulate membrane permeability [187], loading, and on-demand release of contents. Concurrently, changing the physicochemical properties of the vesicle crowns [188] can significantly influence circulation time in vivo, reduce cytotoxicity, and modulate immune responses [189,190]. For example, to improve the low permeability of the polymer vesicles to the enzyme substrate, Tomoki et al. [189] manipulated the surface of the polymer vesicles to make it negatively charged, thus achieving the preferential penetration cationic and neutral compounds, and accelerating the infiltration of the cationic substrate. Furthermore, with advances in the understanding of PD and its treatment requirements, environmentally responsive polymer vesicles, such as those sensitive to pH or temperature, have been developed [188,189], providing new ideas for PD treatment. A polymeric vesicle system that can reversibly switch between monomer/vesicle states according to the cooling/heating cycle was reported [188], and this system can also achieve size adjustability by self-assembly of a thermo-responsive pullulan-graft-poly(propylene oxide) amphiphilic graft copolymer, with great potential for further clinical applications.

Polymer vesicles can also bind to liposomes, overcoming the limitations of liposomes and combining the positive properties of both components. For instance, a novel lipopolymer hybrid nanovesicle system was constructed using CS and a pH-responsive oleylamine-based zwitterionic lipid (OLA) as carriers for vancomycin encapsulation. In this system, the synthesized OLA was used as oleylamino zwitterionic lipid. The dose-dependent toxicity of oleylamine was mitigated, and the vesicle surface was positively charged. This modification enhanced antimicrobial efficacy and promoted targeted drug delivery to the lesion site at low pH value, providing a new strategy for the solution of antimicrobial resistance [191]. In addition, the antibiotic encapsulated polymer vesicles can also be functionalized with nanoparticles, such as cerium dioxide (CeO_2_), to enable multifunctional treatment combining antibacterial and antioxidant activities [192]. However, significant challenges must be overcome before polymer vesicles can be routinely developed for clinical treatment of PD. These include that degradation products are non-toxic and do not cause side effects such as immune reactions, while retaining bioactivity. Additionally, their mass production and improved function must be addressed.

Building on the discussion of local drug delivery systems, vesicle systems represent a class of multifunctional platforms for periodontal therapy. Liposomes exhibit favorable biocompatibility, tissue penetration, and drug encapsulation versatility [74]; however, they are hindered by limited stability, short half-life, and high costs, making them suitable for targeted, short- to mid-term delivery [156]. Exosomes and OMVs provide intrinsic antimicrobial or anti-inflammatory properties and potential cell-specific targeting [163,169]. Nevertheless, variability in cargo, stability issues, and safety concerns limit clinical application. Polymer vesicles demonstrate robust mechanical stability, tunable chemistry, and controlled release capacity, ideal for sustained, multifunctional local therapy; however, their mass production and improved function must be addressed [181]. This comparison clarifies their distinct advantages and limitations, guiding selection based on specific therapeutic objectives.

## 5. Conclusions and Outlook

Local drug delivery systems have shown considerable promise in the treatment of PD by delivering targeted antibacterial agents directly to affected sites. A variety of local drug delivery systems, including fibers, strips and films, microspheres, gels, nanoparticles, and vesicle systems, have significantly improved localized drug delivery while minimizing systemic side effects. These systems provide numerous benefits, including sustained drug release, reduced dosing frequency, and improved patient compliance, making them effective adjuncts to conventional mechanical therapy.

Despite these advances, several limitations remain. Certain traditional delivery systems, such as fibers, are difficult to apply and may require multiple visits for placement and removal. Although strips and films can be adapted to the periodontal pocket, they frequently suffer from insufficient retention. Therefore, the development of improved adhesive technologies and materials is essential to enhance their stability within periodontal pockets. In addition, nanoparticle-based systems may involve uncertain metabolic pathways and potential cytotoxicity, highlighting the need for comprehensive biocompatibility assessments and long-term safety evaluations.

In conclusion, the future development of local drug delivery systems for periodontitis should focus on enhancing retention in periodontal pockets, ensuring long-term biosafety, and integrating multifunctional therapeutic strategies that combine antibacterial, anti-inflammatory, and regenerative effects. Further studies should aim to improve the biodegradability, biocompatibility, personalized design, and affordability of these systems, alongside the exploration of novel materials and fabrication technologies. Specifically, research should focus on polymers and smart materials that provide stimuli-responsive drug release, tailored to individual patient needs. Particular attention should be given to the development of multifunctional local drug delivery systems that can simultaneously deliver antibacterial, anti-inflammatory, and regenerative agents through advanced co-delivery strategies to optimize therapeutic outcomes. Additionally, the creation of environmentally responsive hydrogels and nanoparticles with sustained drug release properties will further advance this field and may offer innovative solutions for effective treatment of PD. Nevertheless, successful clinical translation will require standardized manufacturing processes, rigorous biosafety evaluation, and well-designed clinical trials to validate the long-term safety and efficacy of these systems.

## Figures and Tables

**Table 1:** Advantages and limitations of some common local drug delivery systems for the treatment of periodontitis.

Local Drug Delivery System	Advantages	Limitations
Fiber	Mimic extracellular matrix environment; good biocompatibility and mechanical strength; high porosity; high surface area to volume ratio	Complex operation; may cause redness and swelling of the gums; high production cost limiting clinical translation
Strips and films	Adapt to the shape and size of the periodontal pocket; wider than fibers and more suitable for larger periodontal pockets	Poor retaining ability; some systems are non-degradable and require a second procedure for removal
Microspheres	Protect unstable drugs before and after administration; enable controlled drug release; provide continuous therapeutic effects; improve bioavailability and patient compliance	Limited practical applications in clinical settings
Gels	Flexible; injectable; adjustable mechanical properties; can improve the targeting of loaded drugs	Many hydrogel preparation processes have low efficiency; still far from widespread clinical application
Nanoparticles	Ultra-small size; high surface area to volume ratio; good adsorption capacity; structural stability; targeting capability	Complicated synthesis process; high cost; possible cytotoxicity of metal nanoparticles; unclear metabolic pathways for some nanoparticles
Vesicle systems	Bilayer structure; targeting capability; good tissue permeability	Poor stability; susceptibility to environmental conditions; high cost

**Table 2:** Common polymers used in periodontal local drug delivery systems.

Polymer	Key Physicochemical Properties	Biological Characteristics	Advantages	Representative Applications	References
Polylactic acid (PLA)	Good mechanical strength and structural stability; suitable for electrospinning and core–shell; hydrophobic	Biodegradable and biocompatible	Enables controlled drug release and stable scaffold structures	Nanofibers	[40,41]
Poly(caprolactone) (PCL)	High mechanical strength and slow degradation rate; hydrophobic	Biocompatible and chemically stable	Suitable for long-term drug release and structural scaffolds	Nanofibers and Janus nanofibrous membrane	[42,43,44,45]
Poly (lactic-co-glycolic acid) (PLGA)	Tunable degradation and drug release kinetics; hydrophobic	Biocompatible and biodegradable	Widely used for sustained drug delivery and controlled release	Nanofibers and Janus nanofibrous membrane	[42,45,46]
Chitosan (CS)	Flexible polymer capable of forming gels and films; hydrophilic	Intrinsic antibacterial activity and strong tissue adhesion	Improves local retention and enhances antibacterial activity	Hydrogels	[47,48,49]
Oxidized dextran (OD)	Crosslinkable polymer enabling hydrogel formation; hydrophilic	Responsive to biological stimuli	Enables responsive drug delivery and multifunctional therapy	Hydrogels	[49,50]
Hyaluronic acid derivatives	Injectable hydrogel formation with tunable rheological properties; hydrophilic	High biocompatibility and bioactivity	Promotes tissue regeneration and improves drug retention	Hydrogels	[51]
Cellulose derivatives (cellulose acetate, CA; hydroxypropyl methyl cellulose, HPMC)	Good film-forming ability and mechanical stability; hydrophilic	Biocompatible and mucoadhesive	Suitable for sustained local drug delivery in periodontal pockets	Nanofibers and films	[40,52,53]

## Data Availability

No new data were created or analyzed in this study. Data sharing is not applicable to this article.

## Abbreviations

The following abbreviations are used in this manuscript:

Aa	*Aggregatibacter actinomycetemcomitans*
AMP	ampicillin
aPTT	antibacterial photothermal therapy
aPDT	antibacterial photodynamic therapy
BMP 2	bone morphogenetic protein 2
BP	benzoyl peroxide
BP NS	polydopamine (PDA)-black phosphorus nanosheets
BS	bleach shellac
CEO	clove essential oil
CGA	chlorogenic acid
CHX	chlorhexidine
CMC	critical micelle concentration
CPP	calcium polyphosphate
CS	chitosan
CSNPs	chitosan nanoparticles
Cur	curcumin
DH	doxycycline hydrochloride
DNA	Deoxyribonucleic acid
DSPE-mPEG_2000_	1,2-distearoyl-sn-glycero-3-phosphoethanolamine-N-[methoxy(polyethylene glycol)-2000]
DXY	doxycycline
EVs	Extracellular vesicles
GBR	guided bone regeneration
GMS	glycerol monooleate
GT	green tea extract
GTR	guided tissue regeneration
GTTG	thermoreversible gel of green tea extract
H_2_O_2_	hydrogen peroxide
HPMC	hydroxypropyl methyl cellulose
KA	Kojic acid
MB	methylene blue
Met	metformin
MIN	minocycline
MNZ	metronidazole
Mox	moxifloxacin hydrochloride
MSNs	mesoporous silica nanoparticles
NIPS	nonsolvent-induced phase separation
NIR-II	near-infrared region II
NM	nanostructured barrier membranes
NMP	N-methyl-2-pyrrolidone
OD	oxidized dextran
OLM	oleylamine
OMV	outer membrane vesicles
OTC	oxytetracycline hydrochloride
PAA	poly (acrylic acid)
PBA-PEI	poly (ethylene imine)
PCANs	Proanthocyanidins
PCL	poly(caprolactone)
PD	periodontitis
PDA	polydopamine
PEG	polyethylene glycol
PEO	poly(ethylene oxide)
PG	peptidoglycan layer
PIA	poly (itaconic acid)
PLA	polylactic acid
PLGA	poly (lactic-co-glycolic acid)
PS	photosensitizer
PVA	polyvinyl alcohol
PVP	polyvinylpyrrolidone
(R,S)-PHB	poly[(R,S)-3-hydroxybutyrate]
SDT	Sonodynamic therapy
SFs	strips and films
Sg	Streptococcus gordonii
SRP	scaling and root planing
TH	tetracycline hydrochloride
TMC	N,N,N-Trimethyl chitosan
VM	vancomycin
ZIF-8 NP	framework-8 nanoparticle
